# Searchlight: automated bulk RNA-seq exploration and visualisation using dynamically generated R scripts

**DOI:** 10.1186/s12859-021-04321-2

**Published:** 2021-08-19

**Authors:** John J. Cole, Bekir A. Faydaci, David McGuinness, Robin Shaw, Rose A. Maciewicz, Neil A. Robertson, Carl S. Goodyear

**Affiliations:** 1 Institute of Infection, Immunity and Inflammation, University of Glasgow, Glasgow, G12 8TA Scotland, UK; 2 GLAZgo Discovery Centre, Sir Graeme Davies Building, 120 University Place, Glasgow, G12 8TA Scotland, UK; 3 Glasgow Polyomics, Wolfson-Wohl Building, University of Glasgow, Garscube Estate, Glasgow, G61 1BD Scotland, UK; 4 Beatson Institute for Cancer Research and University of Glasgow, Garscube Estate, Glasgow, G61 1BD Scotland, UK

**Keywords:** Bulk, RNA-seq, Pipeline, Automation, Visualisation, Exploration, Data, Mining, Transcriptomics, Signatures

## Abstract

**Background:**

Once bulk RNA-seq data has been processed, i.e. aligned and then expression and differential tables generated, there remains the essential process where the biology is explored, visualized and interpreted. Without the use of a visualisation and interpretation pipeline this step can be time consuming and laborious, and is often completed using R. Though commercial visualisation and interpretation pipelines are comprehensive, freely available pipelines are currently more limited.

**Results:**

Here we demonstrate Searchlight, a freely available bulk RNA-seq visualisation and interpretation pipeline. Searchlight provides: a comprehensive statistical and visual analysis, focusing on the global, pathway and single gene levels; compatibility with most differential experimental designs irrespective of organism or experimental complexity, via three workflows; reports; and support for downstream user modification of plots via user-friendly R-scripts and a Shiny app. We show that Searchlight offers greater automation than current best tools (VIPER and BioJupies). We demonstrate in a timed re-analysis study, that alongside a standard bulk RNA-seq processing pipeline, Searchlight can be used to complete bulk RNA-seq projects up to the point of manuscript quality figures, in under 3 h.

**Conclusions:**

Compared to a manual R based analysis or current best freely available pipelines (VIPER and BioJupies), Searchlight can reduce the time and effort needed to complete bulk RNA-seq projects to manuscript level. Searchlight is suitable for bioinformaticians, service providers and bench scientists. https://github.com/Searchlight2/Searchlight2.

**Supplementary Information:**

The online version contains supplementary material available at 10.1186/s12859-021-04321-2.

## Background

Once bulk RNA-seq data has been processed, i.e. aligned and then expression and differential tables generated [1], there remains the essential process where the biology is explored, visualized and interpreted (herein known as EVI). EVI typically culminates in the generation of result figures within a report, thesis, or manuscript.

Due to improved tools for quality control (QC) and alignment (e.g. FastP [2], STAR [3] and Kallisto [4]) and the use of automated pipelines the processing stage is now largely trivial, typically taking bioinformaticians only a handful of hours to complete. Despite the obvious advantages, the use of automated pipelines for EVI is less prevalent. With many choosing a bespoke R based analysis, a process which provides freedom in terms of analysis and visualisation but can often take days and sometimes weeks to complete.

Commercial tools for the automation of EVI (such as ingenuity pathway analysis [5] (IPA) and Partek Flow [6]) are the most widely used and generate a comprehensive range of plots and analysis. Whilst also providing convenient means for users to modify plots. Resultantly, they can reduce the time needed to perform the EVI stage to only a few hours, and so typically trivialize much of the EVI stage.

Freely available tools are however more limited. For example, the two most comprehensive—BioJupies [7] and VIPER [8], do not include typical and often key analysis steps, such as heatmaps or boxplots (or similar) of differentially expressed genes. BioJupies is compatible only with human or mouse experiments and incompatible with experiments with greater than two conditions (such as a time-course, comparison of two drugs against healthy, or a CRISPR knockout with suitable controls). VIPER includes only limited means to explore experiments with greater than two conditions (i.e. a Venn diagram but no formal signature analysis). Most critically, neither tool provides users with a convenient means to visually modify the plots that they produce (e.g. font type, axis labels, plot size, grid types, dot or heatmap colors, scaling, etc.). This is particularly limiting in VIPER, as its outputs are visually inconsistent with each other (i.e. different fonts, grid types, color schemes, etc.).

Consequently, these tools are suitable for a fraction of experimental designs only, can require users to backtrack and perform additional manual analysis—even for simple experiments, and in the non-trivial situation that users wish to modify plots visually, (e.g. to make them consistent with each other, consistent with other non-omic results, consistent with a journals figure guidelines, or to resize to fit optimally into figure space), users can be forced to replot entirely using alternative means such as R. Though both tools offer accessible first pass analysis to non-bioinformaticians, because of these limitations, they often ultimately provide bioinformaticians with little or no time advantage over a manual R (or similar) based analysis.

Herein, we describe Searchlight, a freely available tool that automates the EVI stage of bulk RNA-seq analysis. Searchlight aims to:

1. Automate bulk RNA-seq EVI further than other freely available EVI tools by providing a greater range of analysis and visualizations, being suitable for use with a greater fraction of experimental designs and by providing means for users to modify the plots that it generates.
2. Provide a level of bulk RNA-seq EVI automation that is broadly comparable to commercial tools, thereby providing a freely available alternative.
3. Provide analysis and visualizations generated using R scripts and so fit with the working practices of bioinformaticians who typically use R.

We envisage Searchlight to help bioinformaticians, RNA-seq service providers and bench scientists progress bulk RNA-seq research projects rapidly and with minimal effort, thus freeing up resources for further in-depth analysis or alternative analytical approaches.

## Implementation

### Overview of searchlight

**Fig. 1:**
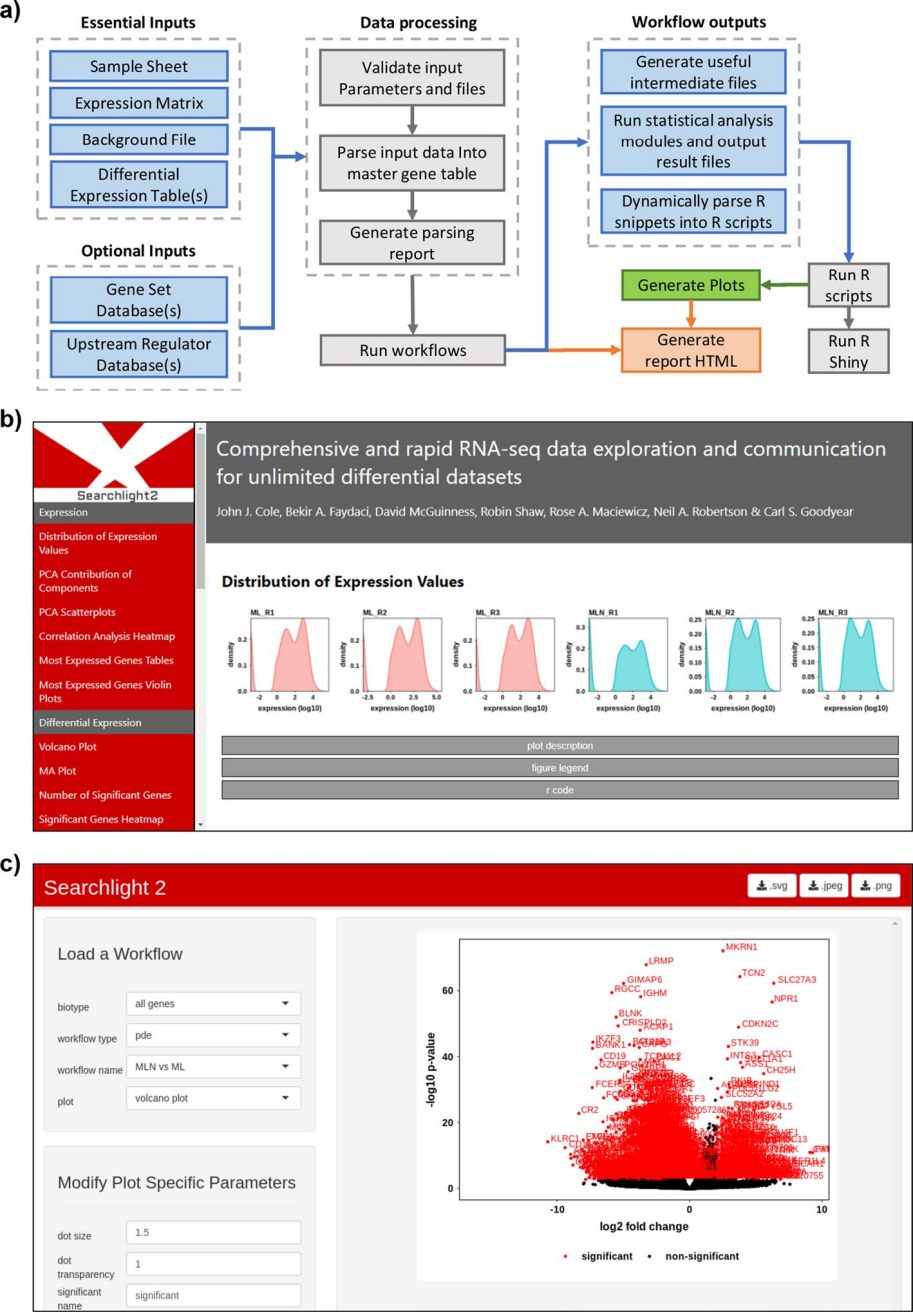
Searchlight outline and screenshots. **a** Searchlight pipeline schematic. Indicating analysis flow (arrows), text file inputs and outputs (blue boxes), plot outputs (green box), report outputs (red box) and processes (grey boxes). **b** Screenshot of a Searchlight report, showing plots, the contents side bar and the plot description, legend and R code drop down menus. **c** Screenshot of the Searchlight Shiny app showing the workflow and plot navigation panel, the plot modification panel and the plot panel

From the outset it is important to note that Searchlight is not a processing pipeline, as it does not perform alignment, count reads or calculate expression and differential expression values. These stages should be completed prior to the use of Searchlight. Any processing method is suitable. Searchlight accepts typical RNA-seq inputs (Fig. 1a), including a tab-delimited sample sheet, matrix of normalized expression values (EM file), genome background file (e.g. as downloaded from Biomart [9]) and any number of differential expression tables (DE file). It is compatible with EM and DE files generated using any method (e.g. DESeq2 [10], EdgeR [11], etc.) or format (e.g. FPKM, TPM, RLog, etc.).

Searchlight is executed as a single command. Firstly, it validates the input files (Fig. 1a) and combines them into a single “master gene table”, from which the downstream analysis is based. Next, it iterates through each workflow generating: intermediate files; statistical analysis result files; per plot and per workflow R scripts, plots; a report in HMTL (Fig. 1b); and a Shiny app (Fig. 1c).

### Workflows

Core to Searchlight is the use of independent but overlapping workflows, that aim to provide compatibility with a broad range of experimental designs. There are three workflows: Normalized Expression (NE), Differential Expression (DE) and Multiple Differential Expression (MDE).

**Fig. 2:**
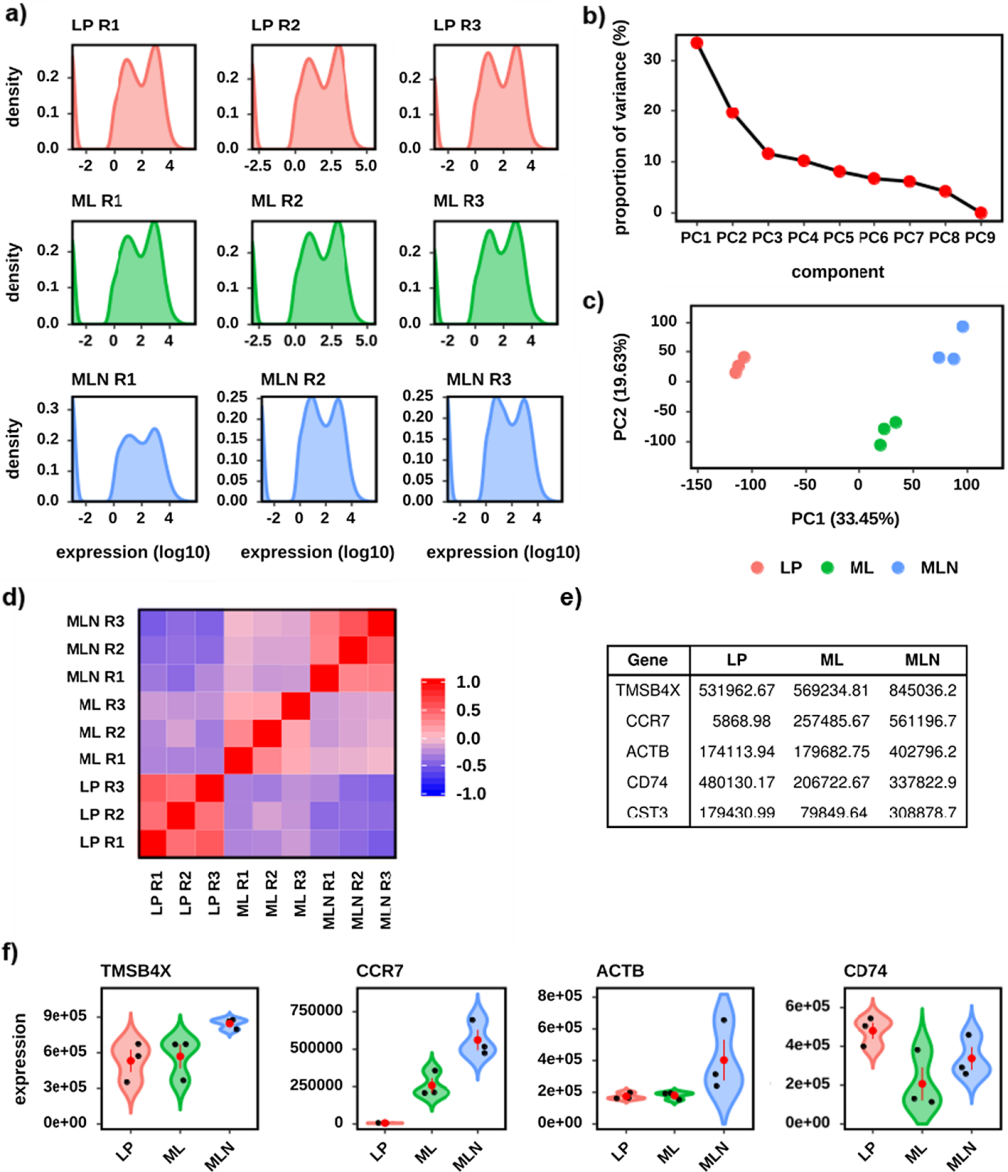
A selection of the default outputs from the Normalised Expression workflow, using the demonstration dataset. Three sample groups are presented—Lamina Propria (LP) (red), Mesenteric Lymph (ML) (green) and Mesenteric Lymph Node (MLN) (blue). **a** Density plots of the per sample distribution of expression values—across all genes. Expression is given on a log10 scale. **b** Principal component analysis (PCA) proportion of variance plot. The % of variation explained by each component is given on the Y-axis. **c** PC1 versus PC2 scatter plot. The % of variation explained by each component is given on the axis label. **d** Sample to sample correlation heatmap. Correlations were determined using all genes and a Spearman Correlation Coefficient (SCC). Colour indicates SCC with − 1 as the darkest blue and 1 as the darkest red. **e** Table of the 5 most highly expressed genes in MLN. Values indicate the mean expression of each gene in each of the three sample groups. **f** Gene expression violin plot with jitter values for each of the four most highly expressed genes in MLN. Black dots denote individual samples. The red dot and whisker denote mean and standard deviation respectively

The NE workflow explores and visualizes the expression data and is focused QC and providing an experimental overview. It includes: expression distribution analysis (Fig. 2a); principal component analysis (PCA) (Fig. 2b, c); distance analysis (Fig. 2d); and highly expressed gene analysis (Fig. 2e, f). See Additional file 1: Table S1 for a full list of NE outputs.

**Fig. 3:**
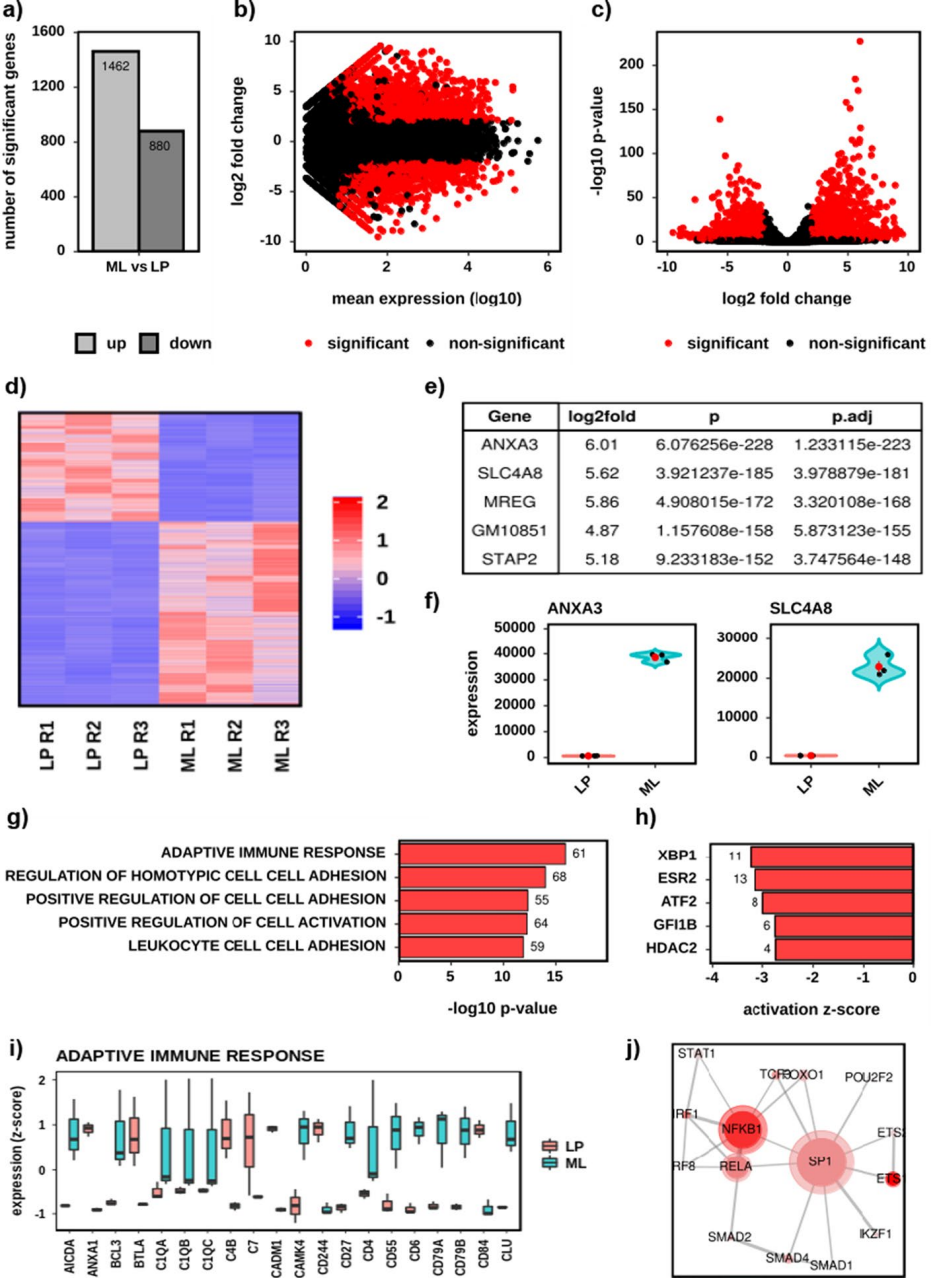
A selection of the default outputs from the differential expression workflow, using the demonstration dataset. Comparison of Lamina Propria (LP) to Mesenteric Lymph (ML). Significance for differential genes was adjusted *p* < 0.01 and absolute log2 fold change > 1. Upregulated genes are higher in ML. **a** Bar chart of the number of up and downregulated genes. **b** MA plot. Significant genes are red and non-significant black. **c** Volcano plot. Significant genes are red and non-significant black. **d** Gene expression heatmap for the 2342 significantly differential genes. Colour denotes row scaled (Z-score) expression values, with darkest blue as lowest expression and darkest red as highest. The Y-axis has been hierarchically clustered using Spearman Correlations, UPMG agglomeration and mean reordering. **e** Table of the 5 most upregulated genes by *p* value. **f** Gene expression violin and jitter plots for each of the two most significantly upregulated genes in ML. Black dots denote individual samples. The red dot and whisker denote mean and standard deviation respectively. **g** Bar chart of the five most enriched gene-sets (GO Biological Processes). The X-axis shows the − log10 *p* value and the data labels the number of significant genes in each gene-set. **h** Bar chart of the five most inhibited upstream regulators (TRRUST). The X-axis shows the activation Z-score and the data labels the number of significant genes associated with each activator. **i** Gene expression boxplots for each gene in the enriched gene-set Adaptive Immune Response. Expression levels are given as per gene Z-scores. Boxes of LP samples are red, and ML are blue. **j** Network plot of the significantly enriched (adjusted *p* < 0.05) upstream regulators. Nodes denote regulators and edges join nodes where > 50% of the regulated genes are shared. Colour intensity represents significance (− log10p) and node size the number of regulated genes

The DE workflow explores and visualizes a single differential expression comparison between two conditions, but can also handle comparisons where a complex linear model was used. It includes: differential gene counts (Fig. 3a); MA plots (Fig. 3b); volcano plots (Fig. 3c); significant gene heatmaps (Fig. 3d), tables with statistical analysis (Fig. 3e) and violin plots (Fig. 3f); spatial analysis (differential gene expression by chromosome); and pathway analysis (Fig. 3g–j) including over-representation analysis (ORA)(e.g. using GO [12], KEGG [13], String [14] etc.) and upstream regulator analysis [5] (URA) (e.g. using TRRUST [15]). For each pathway analysis it explores all, up and downregulated genes separately and plots top hits (Fig. 3g, h), boxplots of gene expression at the top hits (Fig. 3i) and ontology interaction networks (Fig. 3h). See Additional file 1: Table S2 for a full list of DE outputs.

**Fig. 4:**
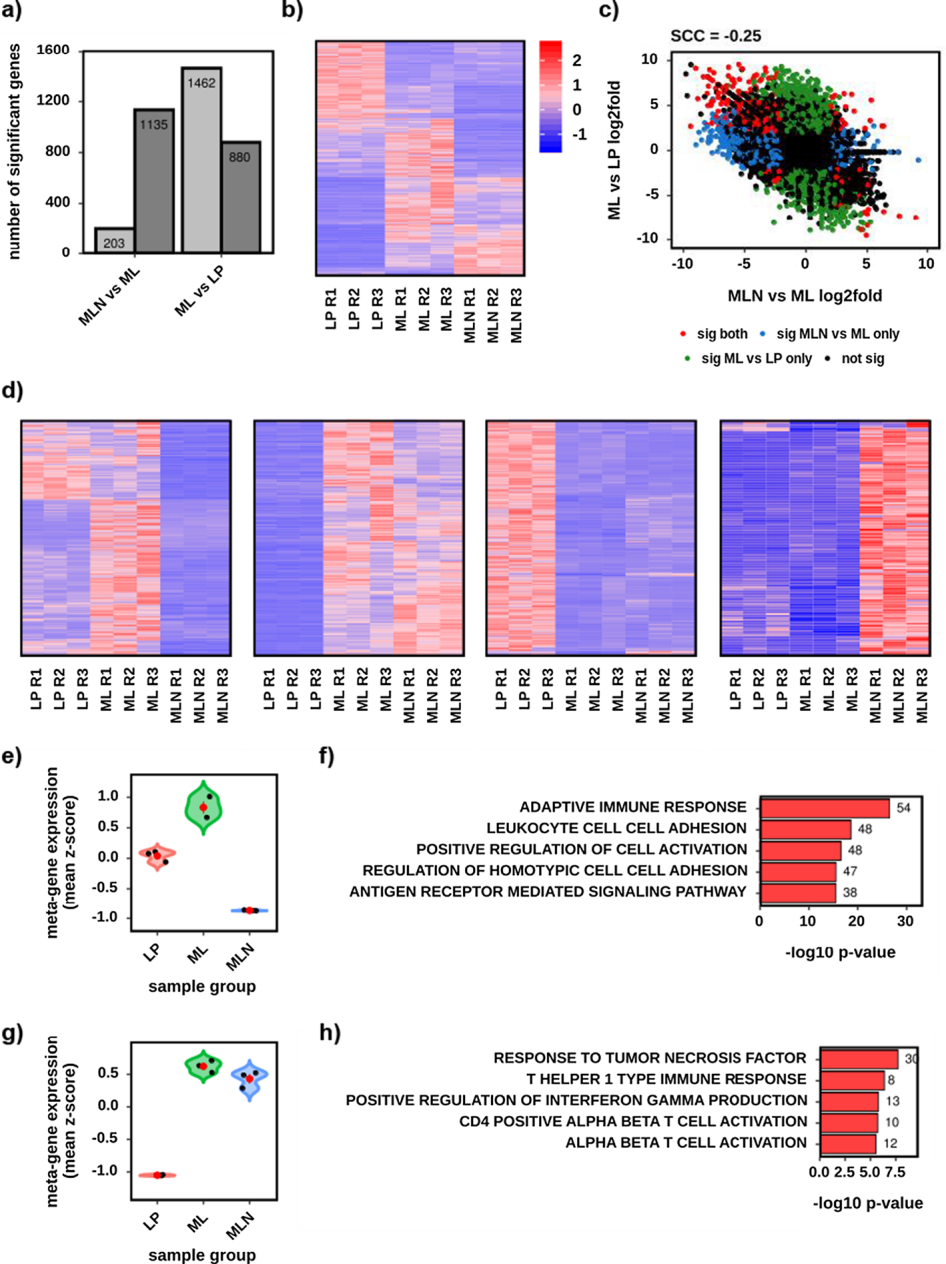
A selection of the default outputs from the Multiple Differential Expression workflow, using the demonstration dataset. Using three sample groups—Lamina Propria (LP) (red), Mesenteric Lymph (ML) (green) and Mesenteric Lymph Node (MLN) (blue), and two differential comparisons ML versus LP and MLN versus ML. Significance for differential genes was adjusted *p* < 0.01 and absolute log2 fold change > 1. **a** Bar chart of the number of up and downregulated genes for each comparison. **b** Gene expression heatmap of the 3,220 significant genes from either comparison. Colour denotes row scaled (Z-score) expression values, with darkest blue as lowest expression and darkest red as highest. The Y-axis has been hierarchically clustered using Spearman Correlations, UPMG agglomeration and mean reordering. **c** Fold versus Fold scatterplot comparing MP versus LP (Y-axis) to MLN versus ML (X-axis) at each gene. Each dot is one gene, with black dots being non-significant, blue being significant in MLN versus ML only, green in ML versus LP only and red in both. The Spearman correlation coefficient (SCC) is − 0.25. **d** Gene expression heatmaps for four of the identified differential expression signatures (1–4). Further plot details are as (**c**). **e** Differential expression signature meta-gene violin plot with jitter values for signature number 1. The mean expression (Z-score) across all genes in the signature is given on the Y-axis. Black dots denote individual samples. The red dot and whisker denote mean and standard deviation respectively. **f** Bar chart of the five most enriched gene-sets (GO Biological Processes) for the genes in signature number 1. The X-axis shows the − log10 *p* value and the data labels the number of significant genes in each gene-set. All gene-sets are significant at *p* < 0.05. **g** As (**e**) however for signature number 4. **h** As (**f**) however for signature number 4

The MDE workflow explores and visualizes the relationship between two or more sets of differential comparisons. For example, it might compare the genes that change between healthy and disease to those that change between disease and disease plus treatment. There is no upper limit to the number of comparisons that can be compared simultaneously with this workflow. It produces analysis and plots such as: significant gene counts (Fig. 4a); heatmaps of all significant genes from any comparison (Fig. 4b); overlap analysis (Venn statistics); fold versus fold analysis (Fig. 4c); and differential expression signature analysis (Fig. 4d–h). For each signature it produces a heatmap (Fig. 4d), meta-gene violin plot (Fig. 4e, g) and ORA top hits plot (Fig. 4f, h). See Additional file 1: Table S3 for a full list of MDE outputs.

Each workflow performs its own set of statistical analysis and generates intermediate files, R code, plots, and a report. Importantly, each workflow may be included any number of times in the same run, and different workflows can be included in any combination. For example, in an experiment with healthy controls (HC), disease (D) and disease with treatment (DT) the user could specify three different DE workflows: HC versus D, HC versus DT and D versus DT, resulting in a separate set of results (and report) for each comparison. The user could additionally specify a single MDE workflow of (HC vs. D) versus (D vs. DT), which would specifically explore the extent to which the treatment reverses the effects of the disease. In this way the user can tailor their analysis to suit the experimental design and research questions.

### Reports

Searchlight collates the results from each workflow into a HTML report, allowing convenient interpretation of results (Fig. 1b). Each report includes all plots alongside descriptions, guides to interpretation, figure legends, methods, and references. The reports also include a hyperlinked contents side bar and cumbersome text sections are hidden within drop-down menus, to help investigators focus on result interpretation.

### Downstream user modification of plots using R

Searchlight uses the R package GGplots2 to generate each plot, and it saves the intermediate data and R script for each outputted plot in the results directory. Resultantly all plots can be conveniently modified and regenerated by altering and re-running these scripts in R. Scripts have a consistent and clear layout with common parameter names. Many visual parameters (such as plot size, font, axis labels, dot colors, etc.) are clearly labelled within each script, and a custom GG theme is used. One script is generated per plot type, as well as a parallel combined script, which can be used to regenerate all plots for a workflow simultaneously. This allows for example the axis font of all plots to be modified together, by modifying only one parameter, once.

### Downstream user modification of plots using a Shiny app

In addition to R scripts Searchlight also generates a Shiny app, which is stored within the results folder. This allows users who are unfamiliar with R to tweak and modify the plots generated by each workflow via an intuitive graphical user interface (GUI) (Fig. 1c). Plots can then be saved to any dimension in jpeg, svg or png format.

### Auto-generated R scripts

When generating each R script during runtime Searchlight utilizes a central bin of 100’s of smaller R-code “snippets”. Each snippet contains code for one small segment of the final scripts. For example, snippets exist for the default theme, plot saving function, default heatmap colors, etc. Searchlight has a master config file, which for each workflow type lists each analysis step. For each analysis step the master config file points to a per-step config file. Examples of per-step config files are ne_PCA_scatterplot and de_significant_genes_heatmap. Each per-step config file lists all the R code snippets required to perform that step, in the order that they should appear in the final R script. During runtime, these snippets are combined dynamically based on the instructions within the master and per-step config files.

Appropriate snippets are shared between scripts. For example, the default theme snippet is used during the generation of every script, whereas the default heatmap colors snippet is used only in those scripts that involve generating heatmaps. Furthermore, some snippets contain tags that indicate where Searchlight should parse certain information (such as *p* value thresholds or sample group names) into the final R script.

### Modifying Searchlight’s default behavior

This system allows users that are familiar with R to modify the default behavior of Searchlight’s plots, by identifying the appropriate snippet and modifying accordingly. By this way users can tailor Searchlight to produce plots of their own visual style by default.

### Analysis modules

Searchlight incorporates several widely used and typical statistical analysis modules:

1. *Over-representation analysis module* This determines enriched gene-sets using a hypergeometric test with Benjamini–Hochberg (BH) correction. It is compatible with any gene-set database (such as GO [12], KEGG [13] and String [14]) provided it is in the GMT format [16]. A selection of databases is included with the software.
2. *Upstream regulator analysis module* This module determines likely activated or inhibited upstream regulators using the method outlined in IPA [5]. It is compatible with any database of upstream regulators, so long as it is in the TRRUST [15] format.
3. *Spatial enrichment analysis module* This module determines expression or differential expression bias at each chromosome in three different ways; bias towards expression, bias towards differential expression, and bias towards up- or down-regulation. All comparisons use a Fishers Exact Test with BH correction.
4. *Overlap analysis module* This module determines the size, enrichment and statistical significance of the overlap between two gene lists, using a Hypergeometric test.
5. *Differential expression signature module* This module generates differential expression signatures based on UPMGA agglomeration. Initially, genes are binned by their differential expression profile (e.g. (A vs. B up), plus (B vs. C up), or (A vs. B up) plus (B vs. C down)). Next, a meta-gene list is generated for each profile, using the per sample median of all per gene z-scores (for each profile). Next, the meta-genes are iteratively merged based on their correlation with each other [Spearman’s Rank Correlation Coefficient (SCC)]. In each iteration the two profile meta-genes of highest correlation are merged, and the meta-gene recalculated. The process continues until no two meta-genes correlate above a given SCC threshold as assigned by the user. The resultant genes in each meta-gene are the differential expression signatures.

## Methods

### RNA-seq processing pipeline

To process raw RNA-seq datasets prior to use by Searchlight we used the following pipeline. Firstly, the fastQ files were QC’d using FastQC [17] (v0.11.7) and then were aligned to the reference genome using STAR [3] (v2.6) with –quantMode GeneCounts –outFilterMultimapNmax 1 and –outFilterMatchNmin 35. For each dataset, we used a Star index with a –sjdbOverhang of the maximum read length − 1. Next, read count files were merged and genes with mean of < 1 read per sample were excluded. Finally, the expression and differential expression values were generated using DESeq2 [10] (v1.24). For differential comparisons we used an A versus B model with no additional covariates, except for re-analysis dataset two (which was paired) where the patient ID was also included. All other parameters were left to default. For the demonstration data (dendritic cell migration) sequences were aligned to the genome and transcriptome GRCm38 (release 93). For the re-analysis datasets sequences were aligned to the genome and transcriptome GRCh38 (release 91).

### Demonstration dataset

To demonstrate Searchlight’s outputs (see “Workflows” section), we used a publicly available bulk RNA-seq dataset (GEO ID: GSE160156) from flow cytometrically-sorted CD103^+^ CD11b^−^ dendritic cells (live, single, CD45^+^, CD64^−^ MHCII^high^ CD11c^+^), that had been acquired from the lamina propria (LP), mesenteric lymph (ML) and mesenteric lymph node (MLN) of C57BL/6 mice (n = 3) under steady state conditions, as previously described [18, 19]. The raw data was processed as described in the RNA-seq processing pipeline section. The dataset was explored using Searchlight (v2.0), specifying two differential expression workflows (see “Workflows” section) (LP vs. ML and ML vs. MLN) and one multiple differential expression workflow [(LP vs. ML) versus (ML vs. MLN)]. Over-representation and upstream regulator analysis were specified using the mouse GO Biological Process [12] and TRRUST [15] databases, respectively. All other parameters were left to default.

### Re-analysis of highly cited datasets

**Fig. 5:**
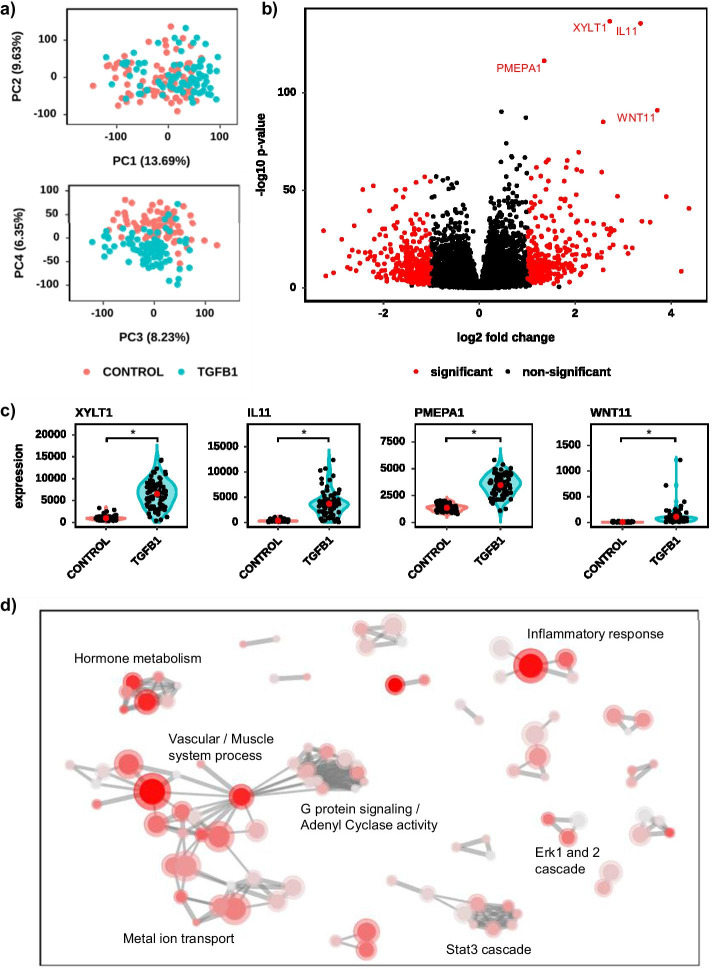
Results from re-analysis of dataset 1 [20] (GEO ID: GSE97358). Comparison between control and TGFB1 treated primary cardiac fibroblasts using Searchlight. Significance for differential expression was adjusted *p* < 0.05 and absolute log2 fold > 1. Upregulated genes are higher in TGFB1 treated. **a** PCA scatter plots, showing PC1 versus PC2 (top plot) and PC3 versus PC4 (bottom plot). The percent variation is given on the axis label. **b** Volcano plot of control versus TGFB1. Significant genes are labelled and in red. **c** Gene expression violin plots for each of the four most significantly differential genes. Significance at *p* < 0.05 and absolute log2 fold > 1 is denoted by an asterisk. Black dots denote individual samples. The red dot and whisker denote mean and standard deviation respectively. **d** Network plot of the enriched (adjusted *p* < 0.05) gene-sets (GO Biological Processes) for the 737 significant genes. Nodes denote gene-sets and edges join nodes where > 50% of the genes are shared. Node colour intensity represents enrichment (− log10 *p* value) and node size the number of significant genes in the gene-set. Representative names for node clusters are given

**Fig. 6:**
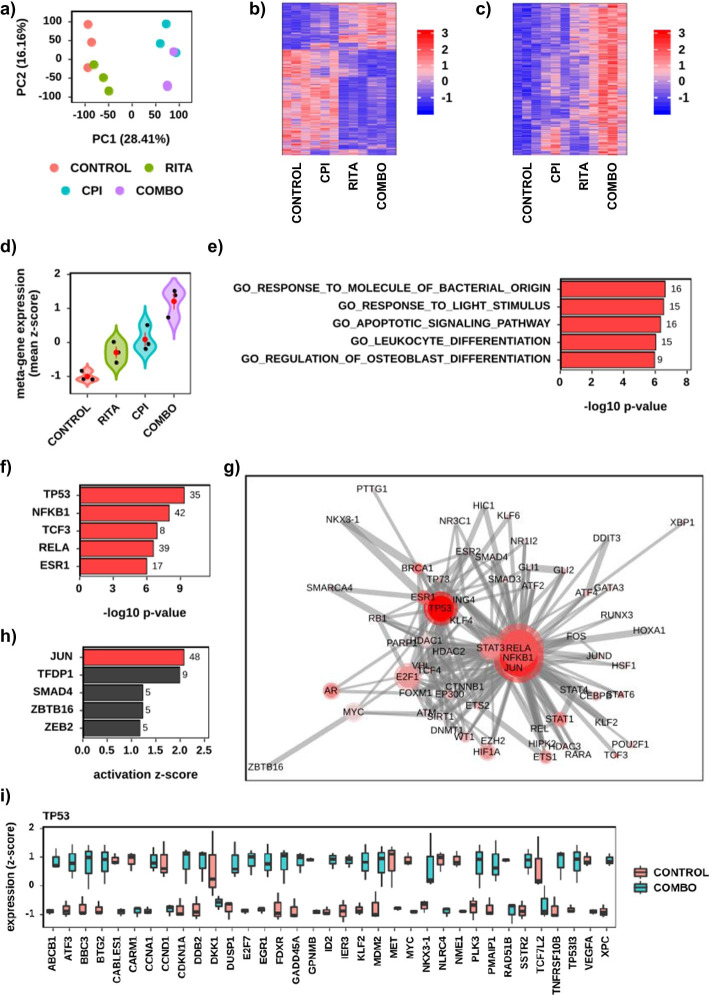
Results from re-analysis dataset 2 [21] (ENA ID: PRJEB9942). Comparison between Control, RITA, CPI, and RITA plus CPI (Combo) treated Chronic myeloid leukemia (CML) haemopoietic stem cells (HSCs). Three differential comparisons were used RITA versus Control, CPI versus Control and Combo versus Control. Significance for differential expression was adjusted *p* < 0.05 and absolute log2 fold > 1. **a** PCA scatterplot showing PC1 versus PC2. The percent variation is given. **b** Gene expression heatmap combining all 2237 significant genes from the three comparisons. Colour denotes row scaled (Z-score) expression values, with darkest blue as lowest expression and darkest red as highest. The Y-axis has been hierarchically clustered using Spearman Correlations. **c** As (**b**) however showing the 329 genes in differential expression signature 4. **d** Differential expression signature four meta-gene violin with jitter values. The mean expression (Z-score) across all genes in the signature is given on the Y-axis. Black dots denote individual samples. The red dot and whisker denote mean and standard deviation respectively. **e** Bar chart of the five most enriched (adjusted *p* < 0.05) gene-sets (GO Biological Processes) for signature four. **f** Bar chart of the five most enriched (adjusted *p* < 0.05) upstream regulators (TRRUST) for Combo versus Control. **g** Network plot of the significantly enriched (adjusted *p* < 0.05) upstream regulators for Combo versus Control. Nodes denote regulators and edges join nodes where > 50% of the regulated genes are shared. Colour intensity represents significance (− log10p) and node size the number of genes that are potentially being regulated. **h** Bar chart of the five most activated upstream regulators (TRRUST) for Combo versus Control. Significantly activated regulators (activation Z-score > 2) are red. **i** Gene expression boxplot for each gene in the enriched upstream regulator TP53. Expression levels are given as per gene Z-scores

To provide example of the utility and time saving features of Searchlight we re-analyzed two highly cited (> 100 citations each) RNA-seq datasets [20, 21] under timed conditions. The bioinformatician was given a raw dataset that they had no previous knowledge of, and they were not permitted web or journal access, or to discuss the dataset. They were given a sample sheet listing sample names and sample conditions but no further information. The bioinformatician was then asked to process, explore, visualize and interpret the dataset, and create a single figure (with multiple panels) that they felt best described the biology. To do so they could use the processing pipeline (see RNA-seq processing pipeline), Combat [22] (for batch correction where appropriate)(v3.38.0) and Searchlight only. As a concession to the one figure limitation investigators were permitted to modify plot sizes and axis text (using R), crop and add data labels where appropriate. They were timed from when they received the raw data and sample sheet, to when the figure as presented in Figs. 5 and 6 was completed. The time spent waiting for the alignment software to run was deducted from the final time. Finally, an alternative investigator then compared the figure to that of the original manuscript, to assess whether the findings had broadly replicated.

Re-analysis dataset 1 [20] (GEO ID: GSE97358) explored the effect of TGFB1 on primary cardiac fibroblasts and had two sample groups (control cells or those treated with TGFB1). The starting point was a table of raw counts, and the investigator (J.J.C.) setup Searchlight for one DE Workflow (TGFB1 versus control) and specified the human GO Biological Process and TRRUST databases for over-representation and upstream regulator analysis respectively. All other parameters were left to default.

Re-analysis dataset 2 [21] (ENA ID: PRJEB9942) explored the synergistic effect of using a combination of RITA, which binds p53 and blocks its degradation, and CPI-203 (CPI), a bromodomain and extra terminal protein (BET) inhibitor on Chronic myeloid leukemia (CML) haemopoietic stem cell (HSC) survival. It had four sample groups Control, RITA, CPI, and RITA plus CPI (Combo). The investigator (J.J.C.) setup Searchlight for six DE (one for each possible combination) and one MDE Workflow ((Combo versus Control) versus (CPI versus control) versus (RITA versus Control)). The initial analysis revealed a strong donor batch effect and so the investigator re-ran the DESeq2 analysis using donor as an additional covariate. In addition, the expression matrix was corrected for the effects of donor using Combat. Searchlight was executed twice—initially using default settings for order and SCC, and then using the order Control + RITA + CPI + COMBO for all workflows (for visualisation purposes) and using a SCC of 1 for greater resolution of the differential expression signatures. The human GO Biological Process and TRRUST databases were used for over-representation and upstream regulator analysis respectively. All other parameters were left to default.

### Comparison to other automated EVI tools

Searchlight was compared, using the OMICtools [23] database as a guide, to other freely available tools for automated bulk RNA-seq EVI. We therefore did not include tools that: were solely focused on the processing stage (e.g. HppRNA [24] and PRADA [25]); had a limited scope for the exploration and visualisation of whole experiments (e.g. QuickRNASeq [26], Consensus Path DB [27], Trapline [28]); were platforms that allow a range of EVI applications but are not inherently automated (e.g. PlotsOfData [29] and Expression Plot [30]); or were platforms for building pipelines but are not necessarily one themselves (e.g. Bioconductor [31] and Galaxy [32]). Having applied these criteria two freely available published tools remained, Biojupies [7] and Viper [8].

We compared Viper, Biojupies and Searchlight under the categories: ease of use, range of compatible experiments, the number and range of outputs (depth of analysis), relevance of analysis, presentation of results and support for downstream modification of plots.

When determining the numbers of outputs per software, we used the following criteria: (1) data that was presented several times only differing in plot parameters were counted only once (e.g. labelled and unlabeled volcano plots); (2) heatmaps of the same data in un-clustered and clustered forms, or using different clustering algorithms were counted once each; and (3) for practical reasons, over-representation analysis visualizations were counted once each regardless of the number of different databases they could be or were used with by default (i.e. we compare the method and visualizations, not the number of databases theoretically available). The outputs of all three tools are summarized in Additional file 1: Table S4.

## Results

### Re-analysis of highly cited datasets

To provide examples of the utility and time saving features of Searchlight we re-analyzed two highly cited (> 100 citations each) RNA-seq datasets [20, 21], under timed conditions. See “Methods” section for full details.

Re-analysis dataset 1 [20] (GEO ID: GSE97358) explored the effect of TGFB1 on primary cardiac fibroblasts and had two sample groups (control and cells treated with TGFB1). The analysis, interpretation, and figure generation (Fig. 5) was completed with 44 min and 30 s of labour from a starting point of raw counts. The PCA (Fig. 5a) showed a clear split between control and TGFB1 treated, which was confirmed by the volcano plot (Fig. 5b), showing 737 differentially expressed genes (adjusted p < 0.05 and absolute log2 fold > 1). The most significantly differential genes were XYLT1, IL-11, PMEPA1 and WNT11 (Fig. 5c). A network of enriched (adjusted *p* < 0.05) gene-sets (GO Biological Process) for the 737 differential genes showed enrichment for Inflammatory Response, Vascular and Muscle System Processes, Hormone Metabolism functions and the role of Erk 1 and 2 signal transduction (Fig. 5d). This replicated the original manuscripts findings that TGFB1 has a profound effect on cardiac fibroblasts expression, with IL-11 and its related pathways as one of the top upregulated genes (see Figure 2 in Schafer et al. [20].

Re-analysis dataset 2 [21] (ENA ID: PRJEB9942) explored the synergistic effects of using a combination of RITA and CPI-203 on CML HSC survival. It had four sample groups Control, RITA, CPI and Combo. The analysis, interpretation, and figure generation (Fig. 6) was completed using 2 h, 37 min, and 11 s of labour from a starting point of raw sequence data. The PCA showed a clear split between all four groups, with PC1 (28%) differentiating samples on CPI treatment and PC2 (16%) on RITA treatment (Fig. 6a). The heatmap of all 2237 significantly differential genes (adjusted *p* < 0.05 and absolute log2 fold > 1) between Control, RITA, CPI or Combo showed CPI to have a much larger effect than RITA (Fig. 6b), and the Combo to reflect the sum of the individual RITA and CPI transcriptional differences. The resultant 329 gene signature from the Combo analysis (Fig. 6c–e), which included BBC3, FOS, FOSB, JUN, JUNB and MDM2, was highly enriched (adjusted *p* < 0.05) for the gene-sets Apoptotic Signaling Pathway, Leukocyte Differentiation and Response to Molecules of Bacterial Origin. Furthermore, in Combo compared to Control, TP53 was the most enriched upstream regulator (adjusted *p* < 0.05) and Jun the most activated (activation z-score > 2) (Fig. 6f–i). TP53 activation was consistent with downregulation of MYC. These observations replicate and expand on the original manuscripts’ findings, that a subset of genes demonstrated extreme synergy. With most genes differentially expressed in response to the combination deregulated in the same direction with RITA or CPI-203. Furthermore, that the combination induced enrichment of TP53 and MYC related pathways (see in Abraham et al. [21] Figure 5 and Extended Data Figures 6 and 7).

In summation, from a starting point of raw data, the bioinformatician was able to broadly recreate the original analysis and conclusions of both datasets (having not previously seen those analysis or conclusions) and present them as figures using under 3 h of labour in each case.

### Comparison to other automated EVI tools

We compared Searchlight to the two tools that currently provide the greatest level of EVI automation—Biojupies [7] and VIPER [8] (Table 1, Additional file 1: Table S4). Note: these also automate the processing step. For full details on selection criteria for comparison see the Methods section.

**Table 1:** Searchlight features and comparison with VIPER and Biojupies

Feature	Searchlight	Viper	BioJupies
Source code available	Yes	Yes	Yes
Type of tool	Command line	Command line	Web
Operating system	Linux/Unix/Windows	Linux/Unix	Any
Computing resources needed (EVI)	1 core, 1 GB RAM	1 core, 1 GB RAM	None
Easy to set up and run	Yes	Yes	Yes—very
Graphical user interface for pipeline	No	No	Yes
Level of automation	Full	Full	Partial
Organism	Any	Any	Human and mouse
Processing pipeline	No	Yes	Yes
EVI pipeline	Yes	Yes	Yes
Intermediate files	Yes	Yes	Yes
Support for complex experimental designs (i.e. more than two different groups of samples)	Yes	Partial	None
Pathway analysis	Over-representation, upstream regulator, differential expression signatures	Over-representation, gene-set enrichment, gene interaction	Over-representation
Total visualisation types (n)	50	20	12
Quality control visualisation types (n)	1	5	1
Expression visualisation types (n)	7	6	2
Differential expression visualisation types (n)	33	5	9
Multiple differential expression visualisation types (n)	9	1	0
Other visualisation types (n)	0	3	0
Plots visually consistent	Yes	No	Yes
Support for downstream user modification of plots	Yes	No	Partial
Graphical user interface for plot modification	Yes	No	Yes
Produces a report	Yes	Yes	Yes
Full report (i.e. descriptions, legends and methods)	Yes	No	Yes

### Ease of use

We found as a web-tool BioJupies the most user-friendly initially, however as it is only partially automated VIPER and Searchlight were more user-friendly once set-up.

### Range of compatible experiments

Being compatible only with human or mouse datasets and only those with two groups of samples Biojupies had the smallest range. Both Searchlight and VIPER had no restrictions.

### Depth of analysis

Overall BioJupies provided the lowest depth of analysis, followed by VIPER, and Searchlight the greatest (Biojupies = 12, VIPER = 20, Searchlight = 50 plot types) (Additional file 1: Table S4). Notably, Searchlight provided 3.7–6.6 times more plot types for differential expression analysis (VIPER = 5, BioJupies = 9, Searchlight = 33), at least 10 times more plot types for multiple differential expression (BioJupies = 0, VIPER = 1, Searchlight = 10), and 2.5–4.2 times more plot types overall. Searchlight was therefore more capable of exploring differential expression and complex experiments than BioJupies and VIPER.

### Relevance of analysis

Overall BioJupies showed the least relevant analysis (Additional file 1: Table S4), uniquely including only a predominantly single-cell relevant clustergram and a thorough small molecules analysis. But having only one type of pathway analysis (ORA). BioJupies had the next most relevant analysis, uniquely including a sample features correlation plot, gene-set enrichment analysis (GSEA) (somewhat a duplication against ORA), gene interaction analysis and the relatively specialized gene-fusion, HLA and virus-seq analysis. Searchlight exhibited the greatest relevance, uniquely including over one of VIPER or BioJupies PCA contributions, PCA beyond component two, sample correlations, an MA plot, significant gene counts, tables of the most differential genes and overlap analysis. Importantly, Searchlight uniquely over both tools included highly expressed gene analysis, heatmaps of differentially expressed genes, violin and jitter plots of the most differential genes, a spatial analysis, boxplots and networks of the most enriched gene ontologies, upstream regulator analysis, fold versus fold analysis and differential signature analysis. Furthermore, Searchlight was more thorough in showing both labelled and unlabeled plot variants (e.g. PCA, Volcano, MA, etc.), clustered and unclustered heatmaps, and all, up- and downregulated genes separately. Searchlight therefore exhibited greater analysis relevance particularly in differential expression and comparison of complex multiple sample groups.

### Presentation of results

We found VIPER to have the least clear presentation of results, notably as visually its plots were not consistent with each other (i.e. font, grids, borders, scaling, dot type, color schemes, etc., differed between plots). BioJupies and Searchlight both had similarly clear and consistent plots. Searchlight had a marginally better presented report, as it included legends (unlike VIPER), a hyperlinked contents bar and it hid cumbersome text.

### Support for downstream modification of plot visuals

VIPER provided no support for downstream modification of plots. BioJupies provided limited support, where users can pre-modify a small number of plot set-up features (e.g. *p* value cut-off, z-score transformation, clustering method, etc.) but none for plot visuals (e.g. font, axis text, dot size, dot type, grids, borders, colors, etc.). Searchlight provided the most support for downstream modification of plots via a Shiny app and standalone per plot and workflow R-code. Both of which were comprehensive.

Over all the criteria Searchlight automated EVI the most comprehensively by some way. Particularly the fraction of experiments it was suitable for exploring, the depth of analysis it provided, and the means for users to modify and tweak plots downstream.

## Discussion

To date, most freely available pipelines for the automation of bulk RNA-seq focus on the processing step, to a greater extent than the downstream EVI [8, 23–28, 32]. To our knowledge, Searchlight is the first freely available, fully automated pipeline aimed exclusively at the downstream EVI step. Though the use of pipelines for automation of the processing step is widespread [1], it is less prevalent for the EVI step. With many researchers favoring at least partly manual methods, such as R. For example, of the 100 most recent (1st May 2021) bulk RNA-seq datasets on the Gene Expression Omnibus (GEO) [33], that had a linked manuscript (for which our institution had access), only 8 cited a commercial or freely available EVI pipeline. Whereas 70 cited R or an R package. The more comprehensive EVI pipelines such as VIPER, BioJupies, Galaxy, Web Gestaldt and IPA, though highly cited (64, 87, 5048, 1684 and 2463 citations respectively), can only account for the analysis of a small fraction of the 156,493 RNA-seq datasets on GEO alone.

The scope for greater application of automated EVI methods is likely considerable. GEO reports 40,588 bulk RNA-seq datasets deposited in 2020. Assuming a similar ratio to the most recent 100 datasets, roughly 28,412 of these datasets were at least partly manually analyzed. Though it’s impossible to precisely gauge the time used to explore, visualize and interpret these datasets, our experiences are that typically this process (up to the point of manuscript figures) takes 2–4 weeks. If we assume conservatively that a bioinformatic researcher costs $25,000 per year, the global burden of manual EVI therefore exceeds 1092 researcher years and $27 million in labour costs, per annum. Thus, EVI remains a major bottleneck in bulk RNA-seq analysis and the underuse of automated EVI pipelines a major unsolved issue in RNA-seq bioinformatics.

The core feature of any automated EVI bulk RNA-seq pipeline is that it should make analysis faster and easier for the user. Thus, ideally it should (1) provide sufficient analysis that users don’t need to perform extensive additional analysis, (2) be compatible with the majority of experiments, organisms and designs users wish to investigate, (3) recognize and allow users to exhaustively change images up or downstream, (4) use files and analysis tools that are familiar to as wide range of users as possible. Accordingly, we have tried to implement all these features within Searchlight.

Searchlight is not a complicated pipeline. Its strength lies in: its range of powerful and widely used analysis and visualization methods; its use of three independent workflows—covering expression, differential expression and signature analysis, that together provide compatibility with a range of experimental designs, whilst also simplifying the analysis; its use of R and R Shiny, as a deliberate attempt to both make it easy to modify visualizations, and appeal to the large number of bioinformaticians who use R.

Consequently, we have shown that Searchlight provides a level of EVI automation that is greater than existing freely available tools. Notably, when compared to VIPER and Biojupies Searchlight produced a 2.5–4.2 greater range of analysis and visualizations, permitted exploration of a greater fraction of experimental designs and unlike VIPER and Biojupies, supported comprehensive up and downstream user modification of plots. Furthermore, we demonstrated that by using Searchlight (alongside a standard Star2 processing pipeline) users were able to re-align, process, explore, interpret, visualize and collate manuscript quality figures that broadly recreated the original analysis, of two highly cited datasets [20, 21], using under 3 h of labour each. Where it is difficult to judge exactly how long this process would take using manual means (it will depend on the dataset, questions, and investigator), it is typically measured in days or weeks. Therefore, our demonstration that it can be completed in a handful of hours represents a reasonable improvement. In a sense this is obvious, as effective pipelining is clearly more time and labour efficient than manual approaches.

Searchlight can provide sufficient analysis to complete small or simple projects (with minor plot tweaks in R), or a comprehensive first pass analysis for larger more complicated projects. Thus, it can help progress research projects rapidly and with minimal effort, freeing up bioinformatic resources for further in-depth analysis, or alternative analytical approaches. Searchlight is suitable for use by bioinformaticians, RNA-seq service providers and bench scientists.

## Conclusions

We have shown that Searchlight automates bulk RNA-seq EVI more completely than the current best freely available tools (VIPER and Biojupies). Providing a 2.5–4.2 greater range of analysis and visualizations, permitting exploration of a greater fraction of experimental designs and organisms, and unlike VIPER and Biojupies, supporting comprehensive user modification of plots. We demonstrated via reanalysis of two highly cited (> 100 citations) publicly available datasets, that it was possible to blindly recreate the original observations in under 3 h of labour. From raw fastQ files to manuscript quality figures. Including all analysis, interpretation and plot tweaking in between. Searchlight therefore provides a rapid and comprehensive alternative to manual R based or current freely available bulk RNA-seq exploration, visualisation, and interpretation methods. Thus, helping free up bioinformatic resources for deeper analytical approaches or additional omic projects.

### Availability and requirements

Project name: Searchlight.

Project home page: https://github.com/Searchlight2/Searchlight2.

Operating system(s): Ubuntu, Windows, Mac OS.

Programming language: Python, R, HTML.

Other requirements: Python, R.

License: MIT.

Any restrictions to use by non-academics: None.

## Supplementary Information

**Additional file 1.Table S1**, a summary list of the normalized expression workflow outputs; **Table S2**, a summary list of the differential expression workflow outputs;**Table S3**, a summary list of the multiple differential expression workflow outputs; **Table S4**, a comparison table of the plot and visualisation types provided by Searchlight, Viper and BioJupies.

## Data Availability

No new datasets were generated as part of this study. The demonstration dataset is available on GEO (GSE160156). Re-analysis dataset 1 is available on GEO [5] (GSE97358) and dataset 2 on the ENA [33] (PRJEB9942).

## Abbreviations

BET: Bromodomain and extra terminal protein
CML: Chronic myeloid leukemia
COMBO: Combination of CPI-203 and bromodomain and extra terminal protein inhibitor
CPI: CPI-203
DE: Differential expression
EVI: Exploration, visualisation, and interpretation
GEO: Gene Expression Omnibus
GSEA: Gene set enrichment analysis
HSC: Haemopoietic stem cell
IPA: Ingenuity pathway analysis
LP: Lamina propria
MDE: Multiple differential expression
ML: Mesenteric lymph
MLN: Mesenteric lymph node
NE: Normalized expression
PCA: Principal component analysis
ORA: Over representation analysis
QC: Quality control
SCC: Spearman correlation coefficient
URA: Upstream regulator analysis
